# Antibiotic prescription for febrile outpatients: a health facility-based secondary data analysis for the Greater Accra region of Ghana

**DOI:** 10.1186/s12913-020-05771-9

**Published:** 2020-10-27

**Authors:** Michael Mireku Opoku, Harriet Affran Bonful, Kwadwo Ansah Koram

**Affiliations:** 1 Department of Epidemiology and Disease Control, School of Public Health, University of Ghana, Legon, Ghana; 2 Department of Epidemiology, Noguchi Memorial Institute for Medical Research, University of Ghana, Legon, Ghana

**Keywords:** Antibiotic prescription, Febrile, Facility-based, Ghana

## Abstract

**Background:**

Misguided prescription of antibiotics is an important contributor towards the emergence and spread of antibiotic resistance. The absence of effective interventions to control antibiotic use leads to increased consumption beyond the needed requirements. Antibiotic stewardship interventions must be appropriately targeted and assessed to enhance the controlled use of antibiotics. The objective of this study was to determine the factors associated with antibiotic prescription to febrile outpatients who seek care in health facilities within the Greater Accra region of Ghana.

**Methods:**

Secondary data obtained from the medical records of 2519 febrile outpatients, consecutively sampled at the outpatient department of 6 health facilities in 3 municipalities during the baseline survey of a quasi-experiment in 2015 was used. The primary outcome was prescription of any antibiotic. Independent variables included patients’ demographics, symptoms, laboratory investigations (blood film microscopy, malaria rapid diagnostic test, full blood count, urine and stool routine examinations), diagnoses, and prescribers’ demographics. Crude and adjusted logistic regression analyses were used to determine the factors associated with antibiotic prescription.

**Results:**

The prevalence of antibiotic prescription was 70.1% (95% CI: 67.7–72.4). Prescribers with more years of practice (> 5 years) were more likely to prescribe antibiotics compared to those with less than 3 years of practice (*p* <  0.001). Integrated Management of Neonatal and Childhood Illnesses (IMNCI) training was associated with a 2.3 (95% CI: 1.54, 3.53, *p* <  0.001) fold odds of antibiotic prescribing. Patients aged 5 years or more were 60% less likely to receive antibiotics compared with those under 5 years (AOR = 0.40, 95% CI: 0.32, 0.51; *p* <  0.001). Patients referred for laboratory investigations were 29% less likely to be prescribed antibiotics than those not referred. The presence of cough as a presenting symptom was associated with a 3.5 (95% CI: 2.54, 4.92) fold odds of antibiotic prescription.

**Conclusion:**

Prescription of antibiotics to febrile outpatients was high. Promoting laboratory testing can potentially reduce irrational antibiotic prescription. Prescribing antibiotics for children under five and the prescribing practices of prescribers with longer years of practice should be targeted with interventions to reduce high use of antibiotics.

## Background

Antibiotic resistance is a major global health challenge. Frequent or misguided consumption of antibiotics are critically important facilitators of the emergence and spread of antibiotic resistance [1, 2]. Higher consumption is not only associated with antibiotic resistance at the individual level but also at the community, national and regional levels, with implications for all patients [2]. A study of the global consumption pattern of antibiotics found that antibiotic consumption increased by 65% between 2000 and 2015 [3]. Consequently, antibiotic resistance is on an upward trajectory [4]. Studies have shown that an increase in antibiotic prescription correlated with an increase in antibiotic resistance [5, 6]. In view of these, the estimate that antibiotic consumption will increase out of proportion to requirements by 2030 if no new policy interventions are implemented is worrying [3].

Developing countries contribute disproportionately more to the increasing trend of antibiotic consumption than developed countries [3, 4, 7]. Owing to the poor availability of data, antibiotic use in these countries is not optimally understood [8]. In African communities, high and inappropriate use of antibiotics have been shown to be widespread [9, 10]. The prevalence of antibiotic prescription in selected hospitals across Africa has been estimated to be 50% [9]. This claims the highest out of all the WHO regions. A comparable point prevalence (51.4%) was determined by an antibiotic use survey at a teaching hospital in Ghana [11]. Inappropriate prescription of antibiotics has also been established among prescribers in other parts of Ghana [12, 13]. The phenomenon is driven by: patients’ demands, poor quality laboratory services, pressure from pharmaceutical companies’ promotional activities, and health worker factors such as non-adherence, and lack of knowledge and lack of access to institutional guidelines [14–16].

Interventions which have been introduced to reduce irrational prescription of antibiotics in Ghana include the Standard Treatment Guidelines (STG) and Essential Medicines List (EML), provision of training and supervision for health care providers and mass education of the public [17]. To ensure rational use of antibiotics and delay potential development of antimicrobial resistance, the Ghana National Policy on antimicrobial use and resistance has been introduced [18].

For these interventions to be successful, the determinants of the prevailing undesirable patterns of antibiotic use need to be identified and appropriately targeted in local antibiotic stewardship education [19]. In the long run, these activities should lead to a reduction in the rate of emergence of bacterial resistance, hospital visits, medical costs and the incidence of side effects [20, 21]. The knowledge gained will be key to informing the policy changes necessary for reversing the increasing trend of antibiotic consumption at all levels.

To these ends, the objective of this study was to determine the factors associated with prescription of antibiotics for febrile outpatients who sought care in health facilities within the Greater Accra region of Ghana using data from the last quarter of 2015.

## Methods

Data for the current study was obtained from the baseline survey of a quasi-experimental study, which sought to develop and assess the effectiveness of a one-way text messaging intervention on health providers’ adherence to malaria case management guidelines [22]. The survey was conducted in 6 health facilities, 2 in each of the 3 municipalities: Ga South, La Dade-Kotopon and La-Nkwantanang Madina, in the Greater Accra region.

In the Ghanaian health care delivery system, patients’ medical records are usually captured in booklets and cards called folders. They are kept on shelves and retrieved manually at every visit by a patient to the health facility. Some health facilities store patient medical information using electronic folders. With the exception of Pentecost Hospital, which operates an electronic patient folder management system, the remaining 5 health facilities (Police, Ga South Municipal and La General Hospitals, Kekele Polyclinic, and Ngleshie Amanfrom Health Centre use the manual system of management of medical information.

A data extraction tool was used to obtain information from the folders of 2519 febrile outpatients in the 6 health facilities between October and December 2015.

It captured information on demographic characteristics, National Health Insurance Scheme (NHIS) membership, presenting symptoms, laboratory investigations requested (including blood film microscopy or malaria rapid diagnostic test, full blood count (FBC), stool examination for parasitic infections (stool routine examination) and simple urine examination for protein and haematuria (urine routine examination), diagnoses and prescribed drugs including information on the dosage regimen. Further to that, the 82 prescribers who saw the outpatients were interviewed using a structured questionnaire. This questionnaire captured information on prescribers’ demographics, exposure to in-service training on malaria case management (MCM) and integrated management of neonatal and childhood illnesses (IMNCI), access to guidelines or other reference materials on MCM, and exposure to supervision in the past 6 months.

Febrile outpatients included in this study were neither detained for observation nor admitted on the ward but treated on ambulatory basis at the outpatient departments. Based on recommendations by the WHO and the STG, being febrile was defined as having a recorded body temperature of 37.5 °C or higher and/or having fever recorded in clinical notes on a survey day [23, 24]. Records of febrile outpatients who were pregnant or who attended for review of previous illnesses were excluded.

### Sampling

Total enumerative sampling was used to select folders of all eligible febrile outpatients daily until the sample size allocated to each municipality was reached. Details on the data extraction tools and detailed methodology of the baseline survey has been published elsewhere [22].

### Data analysis

The entire data obtained from the primary study was validated and analysed using Stata Version 14 [25]. For the purposes of this study, diagnoses of interest were defined as all diagnoses which had a prevalence of at least 5% in the database. Those below 5% were excluded because their frequencies were low, yet, they were so many to have led to overfitting the data, with subsequent unreliable estimates. Some of the diagnoses that were excluded include hypertension, bacteraemia, nephritis, mastitis, and diabetes mellitus. The prevalence of bacteremia and mastitis, for example, were 2.9% (*N* = 2519) and 0.3% (*N* = 2519), respectively.

The outcome variable, prescription of antibiotics, was derived based on whether or not at least 1 antibiotic was prescribed for the patient. A prescribed medicine was considered an antibiotic if it was so classified by the WHO Anatomical Therapeutic Chemical classification, J01 (Antibacterials for systemic use) and P01AB (nitroimidazole derivatives) [26].

The distribution of categorical variables such as sex, the presenting symptoms, the diagnoses and laboratory investigations were assessed using frequencies. For continuous variables such as age, normality was assessed using histograms. To adjust for clustering, unique identifiers for each patient folder and for the municipalities were chosen as primary sampling units and stratum identifiers, respectively. Univariable logistic regression analyses were used to estimate unadjusted odds ratios for all the independent variables. The variables which were significantly associated with antibiotic prescribing (*p* <  0.05) were used to fit a multivariable logistic regression model. Although sex of patient was not significant in the crude analysis it was included in the adjusted model because it was considered a potential confounder. The factors in the adjusted model were checked for multicollinearity using the “Collin” command in Stata 14. A variance inflation factor (VIF) of less than 10 was considered acceptable. There were 1.2% of missing values for patient age and 1.5% for years of practice, totalling 2.7% of the 2519 observations. This was anticipated and adjusted for in the determination of the sample size in the original study. Missing data was addressed using list-wise deletion. Adjusted Wald test was used to determine the significance of multilevel categorical variables in the model. Cluster-Robust standard errors were estimated for all models. Statistical significance was set at *p* <  0.05.

### Ethical considerations

Ethical approval for the primary study was given by the Ethics Review Committee of the Ghana Health Service. Permission was further sought from the Greater Accra Regional Health Administration, the municipal health directorates of the study sites and the management of health facilities and prescribers. Informed written consent were obtained from all the prescribers who saw the patients whose records were used.

## Results

A total of 2519 valid records of patients, who had received care at the selected health facilities in Accra, were used for the study. They were unique singular encounters with patients. Patient and facility level descriptive characteristics are presented (Table 1). Of patients who received antibiotics, 4 out of 10 were aged above 5 years. It must be noted that 1.2% of the folders did not have age recorded in them. The proportion of females who received antibiotics was 51.9%. 57.6% of patients were registered with the main social health insurance scheme in Ghana, the national health insurance scheme (NHIS) or other schemes.

**Table 1:** Facility and patient level descriptive characteristics by antibiotic prescription

	Antibiotic prescription					
	Not Prescribed		Prescribed		Total, ***N*** = 2519	
	N	%^**a**^	n	%^**b**^	n	%^**c**^
**Municipality**						
Ga South (*N* = 811)						
Ga South Municipal Hospital	148	74.7	430	70.1	578	71.3
Ngleshie Amanfrom Health Centre	50	25.3	183	29.9	233	28.7
La Dade Kotopon (*N* = 802)						
La General Hospital	78	35.5	215	36.9	293	36.5
Police Hospital	142	64.5	367	63.1	509	63.5
La Nkwantanang Madina (*N* = 906)						
Kekele Polyclinic	145	43.3	71	12.4	216	23.8
Pentecost Hospital	190	56.7	500	87.6	690	76.2
**Patient age-group**						
< 5	186	24.7	963	54.5	1149	45.6
≥ 5	552	73.3	788	44.6	1340	53.2
Unknown	15	2.0	15	0.9	30	1.2
**Sex**						
Male	343	45.6	848	48.0	1191	47.3
Female	410	54.4	917	51.9	1327	52.7
**Client on NHIS**						
Yes	426	56.6	1026	58.1	1452	57.6
No	327	43.4	740	41.9	1067	42.4

*ABBREVIATION*: *NHIS* National Health Insurance Scheme; a: Proportion of those who received antibiotics; b: Proportion of those who did not receive antibiotics; c: proportion based on total number seen at the municipal facility

The distribution of symptoms of the patients by age group is presented (Table 2). A total of 11,953 symptoms were recorded. While headache (10.5%) was generally the most frequently reported symptom, nasal congestion (11.6%), poor appetite (11.2%), cough (10.3%) and watery stool (8.4%) were more frequently reported among the children under 5.

**Table 2:** Distribution of symptoms by age-group

Symptoms	Age-group in years						
	< 5 (***n*** = 1149)	5–14 (***n*** = 353)	15–29 (***n*** = 435)	30–44 (***n*** = 310)	≥ 45 (***n*** = 242)	Unknown (***n*** = 30)	Total (***N*** = 2519)
**Headache**	392 (7.3)	215 (12.7)	289 (13.1)	201 (14.3)	150 (12.2)	11 (12.0)	1258 (10.5)
**Poor appetite**	600 (11.2)	160 (9.4)	159 (7.2)	95 (6.8)	98 (8.0)	8 (8.7)	1120 (9.4)
**Nasal congestion**	619 (11.6)	140 (8.2)	128 (5.8)	74 (5.3)	68 (5.5)	3 (3.3)	1032 (8.6)
**General Body pains**	360 (6.7)	130 (7.7)	213 (9.7)	152 (10.9)	134 (10.9)	6 (6.5)	995 (8.3)
**Watery stool**	448 (8.4)	126 (7.4)	134 (6.1)	77 (5.5)	74 (6.0)	1 (1.1)	860 (7.2)
**Dizziness**	356 (6.7)	119 (7.0)	176 (8.0)	92 (6.6)	94 (7.6)	4 (4.3)	841 (7.0)
**Cough**	548 (10.3)	94 (5.5)	70 (3.2)	57 (4.1)	47 (3.8)	12 (13.0)	828 (6.9)
**Fatigue**	364 (6.8)	107 (6.3)	160 (7.3)	88 (6.3)	83 (6.7)	2 (2.2)	804 (6.7)
**Dysuria**	366 (6.8)	112 (6.6)	146 (6.6)	80 (5.8)	76 (6.2)	4 (4.3)	784 (6.6)
**Nausea**	354 (6.6)	105 (6.2)	136 (6.2)	72 (5.2)	79 (6.4)	1 (1.1)	747 (6.2)
**Other**	937 (17.5)	389 (22.9)	588 (26.7)	403 (29.0)	327 (100.0)	40 (43.5)	2684 (22.5)
**Total**	5344 (100.0)	1697 (100.0)	2199 (100.0)	1391 (100.0)	1230 (100.0)	92 (100.0)	11,953 (100.0)

Table 3 shows the distribution of diagnoses by age group. On average, 4 (10,026/2519) diagnoses were made per patient. Generally, the predominant diagnosis was acute respiratory tract infection (ARTI) (11.0%). Among the under 5 age group, this pattern of ARTI being the most frequently diagnosed condition was observed (12.7%). However, for all the other age groups, malaria was the most frequently diagnosed condition; 10.7%, 5–14 years; 11.1%, 15–29 years; 10.4%, 30–44 years; 10.4%, ≥ 45 years.

**Table 3:** Distribution of diagnoses by age-group

Diagnoses	Age -group in years						
	< 5 (***n*** = 1149)	5–14 (***n*** = 353)	15–29 (***n*** = 435)	30–44 (***n*** = 310)	≥ 45 (***n*** = 242)	Unknown (2 = 30)	Total (***N*** = 2519)
**ARTI**	618 (12.7)	136 (9.4)	157 (9.5)	101 (9.8)	85 (8.6)	9 (22.0)	1106 (11.0)
**Malaria**	423 (8.7)	156 (10.7)	184 (11.1)	148 (10.4)	103 (10.4)	15 (36.6)	1029 (10.3)
**UTI**	401 (8.3)	119 (8.2)	143 (8.6)	81 (7.8)	71 (7.2)	4 (9.8)	819 (8.2)
**Typhoid fever**	357 (7.4)	113 (7.8)	138 (8.3)	84 (8.1)	79 (7.4)	1 (2.4)	772 (7.7)
**Enteritis**	371 (7.7)	122 (8.4)	127 (7.7)	72 (7.0)	73 (7.4)	1 (2.4)	766 (7.6)
**Pneumonia**	379 (7.8)	103 (7.1)	117 (7.1)	65 (6.3)	68 (6.8)	0 (0.0)	732 (7.3)
**Ear infection**	387 (8.0)	104 (7.2)	113 (6.8)	59 (5.7)	68 (6.8)	0 (0.0)	731 (7.3)
**Tonsilitis**	368 (7.6)	114 (7.8)	114 (6.9)	65 (6.3)	65 (6.5)	0 (0.0)	726 (7.2)
**Anaemia**	344 (7.1)	109 (7.5)	122 (7.4)	70 (6.8)	67 (6.7)	3 (7.3)	715 (7.1)
**Eye infection**	360 (7.4)	107 (7.4)	109 (6.6)	60 (5.8)	66 (6.6)	0 (0.0)	702 (7.0)
**Others**	841 (17.3)	271 (18.6)	331 (20.0)	229 (22.1)	248 (25.0)	8 (19.5)	1928 (19.2)
**Totals**	4849 (100.0)	1454 (100.0)	1655 (100.0)	1034 (100.0)	993 (100.0)	41 (100.0)	10,026 (100.0)

*ABBREVIATION*: *ARTI* Acute Respiratory Tract Infection, *UTI* Urinary Tract Infection

Table 4 presents a summary of the prescriber-patient interactions regarding antibiotic prescriptions at the health facilities during the period when the records were taken. Overall, the prevalence of antibiotic prescription was 70.1% (95% CI: 67.7–72.4%). Age-group stratified analyses showed that prevalence of antibiotic prescription was 83.8% (95% CI: 81.3–86.1) among patients under 5 and 58.8% (95% CI: 55.7–61.9) among patients aged 5 years or more. Of the 1766 prescriptions that had antibiotics prescribed, approximately half (49.2%) were written by physician assistants, 43.9% by medical doctors and 5.7% by nurse prescribers. Regarding the number of years they had been practising, 31.4% of the patients were seen by prescribers who had been practising for less than 3 years, a little over a third (37.7%) had been practising for 3 to 5 years, and 30.9% had been practising for 6 years or more. The largest proportion of the prescriptions with antibiotics were written by prescribers who had been practising for 3 to 5 years (37.4%). While 79.4% of the prescriptions with antibiotics were written by prescribers who had never been trained on integrated management of neonatal and childhood illnesses (IMNCI), 87.9% of the prescriptions without antibiotics were written by those with no training on IMNCI.

**Table 4:** Prescriber-patient interactions by antibiotic prescription

Prescriber Characteristic	Antibiotic prescription					
	Not prescribed		Prescribed		Total, ***N*** = 2519	
	n	%	n	%	n	%
**Prescriber’s Profession**						
Medical Doctor	289	38.4	776	43.9	1065	42.3
Physician Assistant	432	57.4	869	49.2	1301	51.6
Nurse prescriber	24	3.2	100	5.7	124	4.9
Other	8	1.1	21	1.2	29	1.2
Total	753	100.0	1766	100.0	2519	100.0
**Prescriber’s years of practice**						
< 3	279	37.7	500	28.7	779	31.4
3–5	284	38.4	652	37.4	936	37.7
6–9	99	13.4	289	16.6	388	15.7
≥ 10	78	10.5	300	17.2	378	15.2
Total	740	100.0	1741	100.0	2481	100.0
**Ever trained on IM**N**CI**						
Yes	91	12.1	363	20.6	454	18.0
No	662	87.9	1403	79.4	2065	82.0
Total	753	100.0	1766	100.0	2519	100.0

*IMNCI* Integrated management of childhood illnesses.

Medicines prescribed other than antibiotics included antimalarials, analgesics, haematinics, cough remedies, antacids. The proportion of those prescribed antimalarials was 54.2% (408/753) among those who were not prescribed antibiotics and 35.6% (628/1766) among those who were prescribed antibiotics.

A total of 1109 (44.0%) of patients were referred for laboratory investigations, 1007 (40.0%) were tested for malaria, 909 (36.1%) took the full blood count test, 9 (0.4%) took the stool routine examination, and 146 (5.8%) took the urine routine examination. 74.6% of those for whom no laboratory investigation was requested were prescribed with antibiotics. Also, 40.4% of those who received antibiotics were tested.

Results of the multivariable logistic regression analyses (Table 5) identified the following as significant non-clinical factors of antibiotic prescription: location of facility (municipality), age of patient and prescriber’s professional category, years of practice, and training in IMNCI, Compared to prescribers who had practised for less than 3 years, those who had 6 to 9 years of practice experience and those who had practiced for 10 years or more had 3 times (AOR = 2.97; 95% CI: 1.99–4.44) and 1.6 times (AOR = 1.60; 95% CI: 1.12–2.27) higher odds of prescribing antibiotics, respectively. There was no statistically significant difference between antibiotic prescription by clinicians with 3 to 5 years of practice experience and that by clinicians with less than 3 years of practice. Prescribers who had ever been trained on IMNCI had 2.3 times greater odds of prescribing antibiotics than those who had never been trained (AOR = 2.33; 95% CI: 1.54–3.53). After adjusting for all the other covariates, children aged 5 years or above were 60% (AOR = 0.40; 95% CI: 0.32–0.51) less likely to be prescribed antibiotics than those under 5.

**Table 5:** Crude and adjusted logistic regression of factors associated with antibiotic prescribing

	Crude OR (95% CI)	***p***-value	Adjusted OR (95% CI)	***p***-value
**Prescriber’s Profession (Ref: Medical Doctor)**		0.001***		0.029*
Physician Assistant	0.75 (0.60, 0.94)		0.83 (0.58, 1.20)	
Nurse Prescriber	1.55 (1.06, 2.27)		1.30 (0.70,2.41)	
Other	0.98 (0.44, 2.19)		8.33 (1.32, 52.41)	
**Prescriber’s years of practice (Ref: < 3)**		< 0.001***		< 0.001***
3–5	1.28 (0.98, 1.67)		1.12 (0.84, 1.48)	
6–9	1.63 (1.15, 2.31)		2.97 (1.99, 4.44)	
≥ 10	2.15 (1.50, 3.06)		1.60 (1.12, 2.27)	
**Prescriber ever trained on IM**N**CI (Ref: No)**		< 0.001***		< 0.001***
Yes	1.88 (1.38, 2.56)		2.33 (1.54, 3.53)	
**Municipality (Ref: Ga South)**		< 0.001***		< 0.001***
La Dade Kotopon	0.64 (0.49, 0.84)		0.16 (0.09, 0.27)	
La Nkwantanang Madina	1.17 (0.89, 1.54)		0.88 (0.56, 1.40)	
**Sex of patient (Ref: Male)**		**0.235**		**0.627**
Female	0.90 (0.77, 1.07)		1.05 (0.87, 1.27)	
**Age of patient in years (Ref: < 5)**		< 0.001***		< 0.001***
≥ 5	0.28 (0.23, 0.34)		0.40 (0.32, 0.51)	

*P* <  0.05*, *p* <  0.01**, *p* <  0.001***, significance levels; *CI* Confidence Interval, *OR* Odds ratio, *Ref* Reference.

The clinical factors significantly associated with antibiotic prescription as identified from the multivariable logistic regression (Table 6) include laboratory investigation, symptoms including cough, dizziness, difficulty in swallowing, watery stool, and diagnoses including urinary tract infection, typhoid fever, and skin disease. Patients who presented with cough were 3.54 times more likely to be prescribed antibiotics than those who did not present with cough (AOR = 3.54; 95% CI: 2.54–4.92). Patients for whom laboratory investigations were requested were 29% (AOR = 0.71; 95% CI: 0.57–0.89) less likely to be prescribed antibiotics than those for whom laboratory investigations were not requested. Sex of patient, symptoms including general body pains, nasal congestion and diagnoses including ARTI and pneumonia were not significant factors of antibiotic prescribing (*p* <  0.05).

**Table 6:** Crude and adjusted logistic regression of factors associated with antibiotic prescribing

	Crude OR (95% CI)	***p***-value	Adjusted OR (95% CI)	***p***-value
**Cough (Ref: No)**		< 0.001***		< 0.001***
Yes	3.28 (2.57, 4.19)		3.54 (2.54, 4.92)	
**Dizziness (Ref: No)**		0.033*		< 0.001***
Yes	0.81 (0.66, 0.98)		0.48 (0.33, 0.71)	
**Difficulty in swallowing (Ref: No)**		0.001**		< 0.001***
Yes	2.87 (1.51, 5.44)		7.78 (3.43, 17.64)	
**Watery stool (Ref: No)**		0.044*		0.031*
Yes	1.24 (1.01, 1.53)		1.56 (1.04, 2.34)	
**General body pain (Ref: No)**		0.019*		**0.104**
Yes	0.79 (0.65, 0.96)		0.79 (0.59, 1.05)	
**Nasal congestion (Ref: No)**		< 0.001***		**0.110**
Yes	1.67 (1.36, 2.04)		1.30 (0.94, 1.80)	
**Laboratory investigations (Ref: No)**		< 0.001***		0.003**
Yes	0.62 (0.51, 0.74)		0.71 (0.57, 0.89)	
**Pneumonia (Ref: No)**		0.035*		**0.147**
Yes	1.27 (1.02, 1.60)		1.57 (0.85, 2.88)	
**Acute respiratory tract infection (Ref: No)**		< 0.001***		**0.728**
Yes	1.80 (1.44, 2.25)		0.95 (0.70, 1.28)	
**Typhoid fever (Ref: No)**		0.015*		< 0.001***
Yes	1.32 (1.06, 1.66)		3.78 (2.28, 6.26)	
**Urinary tract infection (Ref: No)**		< 0.001***		< 0.001*
Yes	1.51 (1.20, 1.89)		3.53 (2.23, 5.60)	
**Skin disease (Ref: No)**		0.020*		0.020*
Yes	1.54 (1.07, 2.20)		1.82 (1.10, 3.02)	

*P* < 0.05*, *p* < 0.01**, *p* < 0.001***, significance levels; *CI* Confidence Interval, *OR* Odds ratio, *Ref* Reference.

The age-stratified analyses in both crude and adjusted models showed that prescriber training on IMNCI was not significantly associated with antibiotic prescription in patients under 5 years but significantly associated among patients who were 5 years or more (Table 7).

**Table 7:** Crude and adjusted odds ratio of association between prescriber training on IMNCI and antibiotic prescribing

Characteristic	COR	***p***-value	AOR	***p***-value
**Less than 5 years**				
**Prescriber ever trained on IM**N**CI (Ref: No)**				
Yes	1.36 (0.86, 2.13)	0.189	2.05 (0.99, 4.25)	0.055
**5 years or more**				
**Prescriber ever trained on IM**N**CI (Ref: No)**				
Yes	1.93 (1.31, 2.84)	0.001	2.44 (1.47, 4.05)	0.001

*ABBREVIATION*: *AOR*, Adjusted odds ratio, *COR*, Crude odds ratio, *Ref*, Reference

**Fig. 1:**
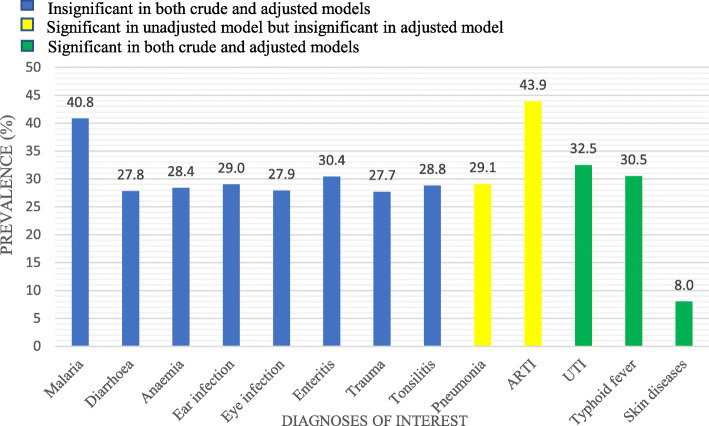
Bar chart showing the prevalence of diagnoses of interest. Diagnoses of interest: all diagnoses which had a prevalence of at least 5% in the database. ARTI, Acute Respiratory Tract Infection; UTI, Urinary Tract Infection

The most prevalent diagnosis among the patients was ARTI (Fig. 1). The diagnoses not found to be significantly associated with antibiotic prescription were malaria, diarrhoeal diseases, anaemia, ear infection, eye infection, enteritis, trauma and tonsillitis (Table 3).

The variance inflation factor (VIF) for the exposure variables in the model were all less than 10 (Table 8), as such none of the variables were considered collinear with any other.

**Table 8:** Variance Inflation Factor for independent variables in the adjusted logistic model

Variable	Variance Inflation Factor
Profession	1.5
Years of Practice	1.1
Prescriber ever trained on IMNCI	1.2
Prescriber sex	1.0
Municipality	1.6
Age	1.3
Cough	1.8
Dizziness	3.9
Difficulty in swallowing	1.1
Watery stool	3.3
General body pain	2.6
Nasal congestion	2.3
Laboratory investigation	1.1
Pneumonia	6.9
Acute Respiratory Tract Infection	1.6
Typhoid fever	4.6
Urinary tract Infection	2.9
Skin infection	1.1

## Discussion

We aimed to determine the factors associated with antibiotic prescription for febrile outpatients. The major factors identified were prescriber’s profession and years of practice, age of patient, presenting symptoms including cough, watery stool, dizziness, difficulty in swallowing, laboratory tests, diagnoses including typhoid fever, urinary tract infection and skin disease.

Other studies have identified acute bronchitis, fever, sputum, tonsillar exudate, patient gender, time spent during prescriber-patient interaction and type of insurance, smoking, rhinorrhoea, sore throat, cough as significant predictors of antibiotic prescription among different populations [27–29]. Others include use of laboratory tests, patient turnout, financing moderated prescribing, medical knowledge and clinical competency, and good clinical practice [30, 31]. Although clinical factors such as symptoms and diagnoses are critical to the decision process for prescribing antibiotics they are rarely investigated for their influence on antibiotic prescription. We discuss our findings with an aim to addressing this gap.

A high prescription of antibiotics for febrile outpatients was observed in our study. The prevalence of prescribing antibiotics, which was 70.1% (95% CI: 67.7–72.4) was higher than was found among febrile outpatients in Zambia [32]. An important difference observed between the 2 studies which could explain the difference in prevalence is that a higher proportion of the febrile outpatients (74.6%) underwent diagnostic testing in the Zambian study than in the present study (44%). Studies in Uganda [33] and Cameroon [30] have shown that laboratory testing reduces the odds of antibiotic prescribing. Our findings showed that when laboratory investigations were requested prior to prescribing, patients were less likely to be prescribed with antibiotics. This was observed although the laboratory tests requested did not include culture test or any non-malaria point-of-care testing for infections. Advocating for the increased use of point-of-care tests for infections could improve the control of antibiotic prescribing.

The prevalence of antibiotic prescription in our study is higher than has been reported by other studies in Ghana, and other developing countries [11, 34, 35]. A critical difference between our study and these other studies is that only febrile patients were included in the current study. The higher use of antibiotics among febrile outpatients may not be justified since a majority of the infections targeted are mostly caused by viruses [36, 37]. It must be stated, however, that our study is limited in further evaluating this as we did not assess the appropriateness of the prescriptions.

Elsewhere, respiratory tract infection has been shown to be predictive of antibiotic prescription [30, 38, 39]. Other studies in Ghana also observed a high prescription of antibiotics for ARTI management at health facilities [40–42]. In the present study, the observation that neither ARTI nor pneumonia is significantly associated with antibiotic prescription seems progressive.

The finding in our study is suggestive of prescribers appreciating that these diseases are more likely to be caused by viruses rather than bacteria [43, 44] or that using antibiotics to treat these diseases provides no clear benefit [45, 46]. This understanding may be translated to the treatment of febrile illnesses at large since these principles apply generally but were not reflected in the findings. Cough is often associated with respiratory infection of one kind or the other. It seems paradoxical that it was found to be significantly associated with antibiotic prescription whilst ARTI was not. Accordingly, a patient who presents with cough is likely to be prescribed an antibiotic, irrespective of whether ARTI or pneumonia is diagnosed. This raises the question of whether prescribers treat symptoms rather than the diseases they diagnose. Another related observation is that patients who present with watery stool are likely to be treated with antibiotics though same cannot be said when diarrhoea is diagnosed. Further studies are required to investigate this practice.

That prescribers with more than 5 years of practice experience are more likely to prescribe antibiotics than those with less experience is at variance with a study conducted in Italy [47] but similar to another study in Canada [48]. Prescribers with fewer years of experience, who ordinarily may not have been promoted to the next level see themselves as amateurs and thus are more likely to adhere to guidelines and probably delay with prescribing antibiotics. However, those with more than 5 years’ experience probably see themselves as experts, working as either a senior prescriber or above the rank of a senior prescriber, so may prescribe based on some experiences. The prescribing practices of more experienced prescribers must be targeted by antibiotic stewardship interventions.

Though inadequate training of prescribers has been shown to worsen antibiotic prescribing [49] we found that those not trained on IMNCI have lower odds of prescribing antibiotics. The subgroup analyses, however, showed that this was the case only among adult patients. Thus, our finding is consistent with findings from other studies that IMNCI training is beneficial for antibiotic prescription in children under 5 [50, 51]. IMNCI is child-focused preventative and curative care implemented in a way as to improve healthcare worker skills, the overall health system, and family and community health practices. It is noteworthy that IMNCI training is not implemented for the management of adult illnesses. It must be pointed out that one approach recommended by IMNCI is the active involvement of patients or caregivers in deciding on treatments [52]. This approach, however, has been suggested as influencing high antibiotic prescribing [29]. In countries such as Ghana where prescribers who attend to children tend to also see adults it may be useful to conduct further studies to investigate the residual influence of IMNCI training on antibiotic prescription in adults.

Regarding age, we found that patients aged 5 years or more had lower odds of being prescribed antibiotics. A similar observation was made in Cameroon [30]. Children under five, as a result of having less developed immune systems are more susceptible to infections than older people. Prescribers are more likely to manage illnesses of children in this age group with antibiotics than in older age groups. Also, the higher rate of mortality among children under 5 may condition prescribers to prefer erring cautiously by prescribing antibiotics given that treatments are usually done empirically. This is occasioned by the resource constraints of the health facilities and relatively expensive diagnostic tests. This trend is worrying considering that high antibiotic consumption in childhood has been linked with the development of metabolic diseases in later life [53].

We conducted a literature search on PubMed and Google scholar, and to the best of our knowledge this is the largest published study on antibiotic prescription in Ghana. The relatively large sample size allowed for the inclusion of several factors in the assessment. One limitation of this study is that the period of the data collection was short (3 months). As such, seasonal variations in antibiotic prescription was not accounted for. Another limitation of this study is the assumption that all documented actions were carried out. Similarly, all undocumented actions were assumed not to have been taken. This may have introduced misclassification bias but was considered more acceptable than direct observation which may have caused prescribers to vary their usual prescribing habit. Also, behavioural factors such as physician perception of patient desire, patient expectation and smoking were not assessed. Finally, findings of this study may not apply to febrile patients who were admitted due to disease severity.

## Conclusion

The factors associated with antibiotic prescription for febrile outpatients were prescriber’s profession and years of practice; age of patient; some presenting symptoms which included cough, watery stool, dizziness, difficulty in swallowing, laboratory tests; some diagnoses including typhoid fever, urinary tract infection and skin disease. In contrast, ARTI and pneumonia were not significantly associated with antibiotic prescription.

The rate of antibiotic prescription for febrile outpatients is high compared to practices from settings with similar burden of communicable diseases. To reduce this, advocacy for the increased use of laboratory testing prior to prescribing antibiotics is key. Also, more experienced prescribers and treatment for children under five should be targeted with interventions aimed at reducing antibiotic prescribing. IMNCI training appears to be counterproductive for controlling antibiotic prescribing in adults.

## Data Availability

The dataset is not publicly available. However, it can be accessed after a reasonable request has been sent to the corresponding author.

## Abbreviations

ARTI: Acute Respiratory Tract Infection
EML: Essential Medicines List
IMNCI: Integrated Management of Neonatal and Childhood Illnesses
NHIS: National Health Insurance Scheme
OPD: Outpatient department
STG: Standard Treatment Guidelines
UTI: Urinary Tract Infection
WHO: World Health Organization
